# Beating Heart Motion Accurate Prediction Method Based on Interactive Multiple Model: An Information Fusion Approach

**DOI:** 10.1155/2017/1279486

**Published:** 2017-10-15

**Authors:** Fan Liang, Weihong Xie, Yang Yu

**Affiliations:** ^1^Tianjin Key Laboratory of Information Sensing & Intelligent Control, Tianjin University of Technology and Education, Tianjin, China; ^2^Case Center for Imaging Research, Case Western Reserve University, Cleveland, OH 44106, USA; ^3^Department of Radiology, University Hospitals Case Medical Center, Case Western Reserve University, Cleveland, OH 44106, USA; ^4^Technology, Humanities and Social Sciences Department, Guangdong University of Technology, Guangzhou, China; ^5^Department of MIS, Marketing and Digital Business, Rochester Institute of Technology, Rochester, NY 14623-5603, USA

## Abstract

Robot-assisted motion compensated beating heart surgery has the advantage over the conventional Coronary Artery Bypass Graft (CABG) in terms of reduced trauma to the surrounding structures that leads to shortened recovery time. The severe nonlinear and diverse nature of irregular heart rhythm causes enormous difficulty for the robot to realize the clinic requirements, especially under arrhythmias. In this paper, we propose a fusion prediction framework based on Interactive Multiple Model (IMM) estimator, allowing each model to cover a distinguishing feature of the heart motion in underlying dynamics. We find that, at normal state, the nonlinearity of the heart motion with slow time-variant changing dominates the beating process. When an arrhythmia occurs, the irregularity mode, the fast uncertainties with random patterns become the leading factor of the heart motion. We deal with prediction problem in the case of arrhythmias by estimating the state with two behavior modes which can adaptively “switch” from one to the other. Also, we employed the signal quality index to adaptively determine the switch transition probability in the framework of IMM. We conduct comparative experiments to evaluate the proposed approach with four distinguished datasets. The test results indicate that the new proposed approach reduces prediction errors significantly.

## 1. Introduction

Robot-assisted motion compensation, as one of the promising treatment modalities for* Coronary Artery Disease* (CAD) related surgery, including* Coronary Artery Bypass Graft* (CABG) Surgery, and* Beating Heart Intracardiac Surgery*, has some expected advantages, such as the relative high accuracy and the possibility of new surgery techniques, for example, minimally invasive beating heart surgery. The off-pump surgery also brings central nervous risk and neurocognitive problems [1]; meanwhile, the surgeon has the limitation of the hand movement bandwidth to track beating heart motion and ensure a high-accurate operation. The surgery-assisted robot system provides a relatively stationary scenario for the surgeon by eliminating the relative motion between the beating heart and robotic tools. Typically, the system needs a high tracking accuracy while in moving status in order of 100–250 *μ*m root mean square (RMS) [2].

Similar to typical object tracking problems, the modeling of the target motion dynamics issue in the beating heart motion compensation procedure is an essential challenge. However, the major difficulty of beating heart motion tracking arises from the target motion uncertainty exhibiting itself in diverse situations where a target may undergo uncertain trajectory pattern during elusive time instants. The beating heart motion results from the autonomic neural regulation of the heart and the circulatory system with variant complex fluctuations [3, 4]. At a normal state, the heart motion regularly beats with respiratory sinus arrhythmia, which demonstrates a distinct phase coupling nonlinearity in its beat to beat pattern [5]. The beating heart motion trajectory from* Coronary Heart Disease* (CHD) patients usually involves arrhythmia as well, such as atrial fibrillation and premature ventricular contraction (PVC), which have abnormal rhythms as a result of the defected cardiac conduction system. In general, the beating heart motion with strong variability dictating its beat to beat trajectory not only in continuously changed heart rate but also in its varied movement patterns.

There are several research groups that devoted numerical efforts [2, 6–11] on the topic of robotics-assisted motion compensation problems.* Proportional-derivative *(PD),* pole placement* (PP), and linear quadratic controllers [7] were first implemented to solve the tracking control problem. However, these controllers are inadequate in the performance due to their phase lag caused by the feedback measurement [2]. In the literature [8, 12–18], researchers proposed higher effective model predictive control approaches by importing the prediction of the future heart motion in the feed-forward loop as in [2, 12, 16, 19].

As a prerequisite for the control strategy to achieve robust and precise position tracking, the heart future movement prediction is widely studied [2, 7]. In general, the current methods could be categorized into (1) method based on nonmodel: the model-free Taken Theory based prediction method [20]; (2) methods based on the linear or nonlinear model: linear model based prediction methods [17, 21], for example, time domain AR model [2] and Fourier Series based prediction method [9, 11]; nonlinear model based prediction method: for example, Saritas [22] proposed a prediction method by using artificial neural network, Bachta et al. [8] proposed an amplitude modulation based prediction method, and Liang et al. [13, 16] described the cardiorespiration coupling by a quadratic nonlinear model; (3) uncertainty compensation method: Liang et al. [14] using a master-slave Kalman filter to dynamically estimate the process noise covariance; and (4) other physiological signal assisted methods: these studies [15, 19, 23] investigated the correlation between ECG and beating heart motion.

To deal with the specific features of rhythm irregularities, Tuna proposed an adaptive motion estimation algorithm. However, the study covers atrial fibrillation arrhythmia condition only, and the other types of arrhythmias like PVCs still need to be validated.

In the above method, intensive studies make considerable efforts on two issues in beating heart motion compensation; one is the nonlinearity, and the other is uncertainty. The two distinct features are dominant in the dynamics of beating heart motion, which hinders the accurate motion prediction. The nonlinearity derived from the coupling of breath and heart beat motion is the dominant mode in the dynamics of the heart rhythm, which shows a small amount of time-variant statistics changing. We also noted that the uncertainty exists in and prevails in some specific situations, which has an extreme unpredictable pattern in the arrhythmias and could not be recognized from the prior knowledge.

In general, the majority methods in the literature have the flaw to cover varying characteristics of beating heart motion. Thus, a signal model could be problematic due to either overfitting or underfitting the actual state under the variations caused by the nonstationary nature and noise.

Although there is no universal model that can characterize the motion of a beating heart, a finite number of models can adequately describe the heart behavior in different regimes [24, 25]. We need to consider both normal heart beating situations and those arrhythmia-related irregularities to improve the prediction accuracy and thus enhance the robot system performance. Therefore, it is clear that purely quadratic nonlinear model could not fully describe the beating heart motion dynamics. Another more general prediction framework, which covers both the normal nonlinearity-dominant beating mode and the abnormal arrhythmia-related irregular beating mode necessary for surgery of each patient with an individual difference, is needed.

In this paper, we propose a* Nonlinear Fusion Adaptive Model* (NFAM) based heart motion prediction method. In NFAM, we employ IMM Kalman filter approach capturing every possible mode of beating heart motions. The hybrid model fuses the dynamics of the modulated sinusoids model [14, 16] to describe the normal nonlinear heart motion mode and uncertainty irregular model [15, 19] to handle various unforeseen arrhythmia patterns, especially for the quick changing pattern of AF and PVCs. The novelty of this method lies in that we import the fusion framework to handle the unpredictable fast time-variant uncertainty in the case of arrhythmia during heart beating as well as maintaining the good prediction performance under regular heart rhythm. The NFAM could enhance the robust ability to deal with the abrupt, unforeseen uncertainties and approximate the motion with sufficient detail.

## 2. Methodology

### 2.1. Beating Heart Motion Characteristics

The heart motion dynamics is a nonlinear, nonstationary process with complex fluctuating. The interpretation of the complicated mechanisms of the cardiovascular system that derives the nonlinearity and uncertainty are diverse, such as multiple oscillators interaction [3], autonomic neural regulation of the heart and the circulatory system [5], and stochastic feedback regulation parasympathetic and sympathetic branch of nerve system [4].

The dynamics of beating heart motion demonstrates two trajectory patterns, the regular one and the irregular one. Although the beating heart motion does not beat with a constant period, it has the relative regular pattern with small time-varying statistics at a normal state. The regular pattern roots from regulation of the heart function showing the fix-chained phases. And there is another important phenomenon called arrhythmia in cardiac physiology, strongly impacting the dynamics of beating heart motion. The most common CHD patients associated arrhythmias are AF and PVCs which would make the motion beat either slower (commonly known as bradyarrhythmias) or faster (commonly known as tachyarrhythmias). AF is one of the most common types of serious arrhythmia, which involves a fast and irregular contraction of the atria. Atrial fibrillation would cause symptoms like a “fluttering” heartbeat or an irregular pulse. The morphology of PVCs is highly variable and relies on the place of origin and the presence of structural heart disease. In addition, the trajectory of PVCs is hard to predict because sometimes the activation spread occurs from a ventricle to the contralateral one through nonspecialized myocardium; however, when activation comes from a fascicle through a specific conduction system, both ventricles could be activated “synchronously.”

The AF and PVCs arrhythmias are with different irregular pattern case by case. After exploration of the physiology nature and observations of the measured trajectory of beating heart motion, we noted the following:

1. The features in each mode: at the regular heart beat state, time-varying cardiorespiration interaction, which has the “fixed” pattern within limited uncertainty range, is the regular dominant pattern running in the beating heart dynamics. At irregular beating state, relatively large variation occurs under arrhythmia such that beating heart motion undertakes a huge alteration from normal beat and transits into another mode that we could not determine accurately due to the insufficient knowledge of the underlying mechanism.
2. The overall states feature: the states of beating heart dynamics are neither only “fixed,” demonstrating the regular beating, nor only the “chaos,” demonstrating the irregular beating. The states are merging the two modes characterized by being sometimes dominated by “fixed” mode and sometimes dominated by “chaos” mode, and others are something in between.

### 2.2. Nonlinear Fusion, Adaptive Model of Beating Heart Motion

#### 2.2.1. Nonlinear Adaptive Model

The normal state dynamics of the beating heart motion is a process driven by coupled respiration motion and cardiac motion, each with individual characteristic frequency. Coupled oscillators interact with each other through their amplitudes and phases [16]. Therefore, we approximate the beating heart motion by the superposition of three parts: cardiac motion *X*_*c*_, respiration motion *X*_*r*_, and interaction motion part *X*_*m*_. The model could be represented as

(1) $$\begin{array}{c} X\left(\;t\;\right)=X_{c}\left(\;t\;\right)+X_{r}\left(\;t\;\right)+X_{m}\left(\;t\;\right) \end{array}$$

in which

(2) Xct=∑k=1Ncakccos⁡2πfkct+ϕkc

(3) Xrt=∑l=1Nralrcos⁡2πflrt+ϕlr

(4) Xmt=Xc·Xr=∑k=1Nc1 ∑l=1Nr1aklmcos⁡2πfklmt+ϕklm.

In (2)–(4), generally *a* denotes the magnitude, *f* denotes the frequency component, and *ϕ* denotes the phase. Equations (2) and (3) are the linear combinations of the significant cardiac harmonics and respiration harmonics, respectively. Equation (4) is the quadratic term with meaning of modulating the cardiac signal by respiration signal.

The state space model for this system is

(5) xt+Δt=AΔtxt+μtzt=hxt+vt

with state vector defined as *x*(*t*) = [*c*(*t*), *a*_*k*_^*c*^(*t*), *a*_*l*(*t*)_^*r*^, *a*_*kl*(*t*)_^*m*^, *f*^*c*^(*t*), *f*^*r*^(*t*), *f*^*m*^(*t*), *θ*_*k*_^*c*^(*t*), *θ*_*l*_^*c*^(*t*), *θ*_*l*_^*r*^(*t*), *θ*_*kl*_^*m*^(*t*)]^*T*^ Here the transfer matrix *A* could be derived if the variable *c*(*t*) and all *a*(*t*), *f*(*t*), and *ϕ*(*t*) could evolve through a random walk.

(6) $$\begin{array}{c} A\left(\;\Delta t\;\right)=\left[\begin{array}{cccc} 1 & & & \\ & I_{N_{c}+N_{r}+N_{r1}} & & \\ & & A_{f}\left(\Delta t\right) & \\ & & k\Delta t & \\ & & \vdots & \\ & & l\Delta t & A_{\theta }\left(\Delta t\right)\\ & & \vdots & \\ & & N_{r1}\Delta t & \end{array}\right], \end{array}$$

where *A*_*f*_(Δ*t*) and *A*_*θ*_(Δ*t*) hold the same structure to represent the coupling feature of the heart motion.

(7) $$\begin{array}{c} A_{f}\left(\;\Delta t\;\right)=\left[\begin{array}{ccccc} I_{N_{c}} & & & & \\ & & I_{N_{r}} & & \\ 1 & & 1 & & \\ 1 & & & 1 & \\ & 1 & 1 & & 0_{N_{c1}}\\ & 1 & & 1 & \end{array}\right]. \end{array}$$

Also the *h*(*x*(*t*)) could be formulated as

(8) HTδhδxTx^t+Δt=Ax^t=1cos⁡θ^1ct+Δt⋮cos⁡θ^klmt+Δt⋮−a^kct+Δt ∣ tsin⁡θ^1t+Δt⋮−a^klmt+Δt ∣ tsin⁡θ^mt+Δt.

#### 2.2.2. Uncertainty Irregular Model

In this mode, we describe the irregular beating heart motion as the combination of adaptive linearity. Specifically, first, we model the main part of the arrhythmia via autoregressive (AR) model. Second, we loosely chose the process noise covariance due to the significant uncertainty existing in the irregular pattern. The reason we choose the AR to model the beating heart motion arises from the following facts. The underlying electrophysiological mechanisms of arrhythmia are very complex and are affected by a multitude of factors [26]. It is not practical to construct in vivo models of arbitrary arrhythmia types. After some experiments, we found that the frequency spectrum of two kinds of arrhythmias derived by FFT algorithm matches the spectrum generated by high-order AR model. Furthermore, the former study [27] which adopts the AR model shows successful results that if the behavior of the heart changes abruptly especially under the arrhythmia situations, the predictors can adapt the new heart behavior and can track the ideal time-varying solution.

(9) x⃑k=A1x⃑k−1+A2x⃑k−2+⋯+Apx⃑k−p+v⃑k.

Each observation is given by sum of weighted past *p* observations along with the zero-mean white noise $\overset{⃑}{v}(k)$. We also take the *p* as the order of the AR model.

The state space canonical form of the UIM could be reformulated as

(10) xk=Fxk−i+Bvkyk=Cxk+nk.

*F* is the canonical form of dimensions *p* by *p*:

(11) $$\begin{array}{c} F=\left[\begin{array}{cccc} A_{1} & A_{2} & \cdots & A_{p}\\ I & 0 & \cdots & 0\\ 0 & I & & \\ & & \ddots & 0 \end{array}\right]. \end{array}$$

In algorithm design, we select the two different model orders of 8 and 12, respectively, to adapt diverse and complex irregular situations. We name the model as UIM8 and UIM12, respectively.

#### 2.2.3. Fusion Method

The biggest challenge of beating heart motion prediction arises from the target motion uncertainty. The conventional solutions to the prediction problems in beating heart surgery follow the strategy that can be characterized as “firstly build a parameter model as accurate as possible and then adaptively estimate the parameters to implement the model, which approximate the trajectory [28].” This approach has some drawbacks: (1) possible errors in the decision on the model are not likely to be accounted in the estimation process and (2) the determined single model is not able to account for all possible situations of the right trajectory. The Interactive Multiple Model (IMM) based prediction method reduces the difficulty due to model uncertainty by using more than one model, which the models interplay with each other to achieve better performance. The conventional algorithm relies entirely on the performance of one single “best” model decided by the prior knowledge of the target motion. In contrast, the IMM call all the individuals (models) in a group simultaneously to produce overall estimation.

**Figure 1:**
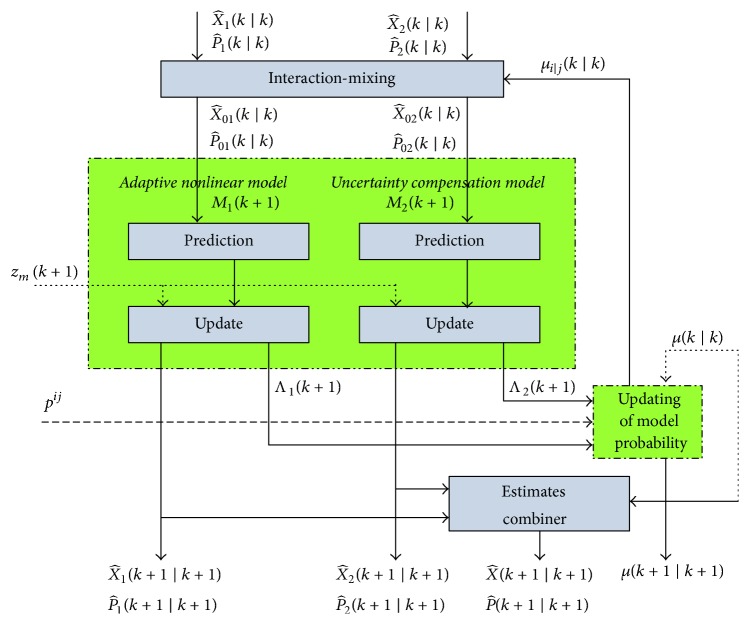
Diagram of beating heart motion prediction algorithm based on IMM.

The fusion method diagram is illustrated in Figure 1.

In the NFAM approach, IMM uses two distinct possible models simultaneously; the first one is an adaptive nonlinear model, and the other is irregular uncertainty model. In addition, IMM employs a probabilistic switching mechanism for exchanging of some information between the filters in the fusion task [29]. We unify the evolving target motion and measurement as the following equations:

(12) Xk+1=FjXk+wjkzk+1=HjXk+1+vjk+1.

*(a) Interaction and Mixing.* In the mode *M*_*j*_(*K* + 1), the mixed estimate *X*_0*j*_(*k*∣*k*) and the covariance matrix *P*_0*j*_(*k*∣*k*) in each cycle are computed as

(13) X^0jk ∣ k=∑i=1rμijk ∣ kX^ik ∣ kP^0jk ∣ k=∑i=1rμijk ∣ kP^ik ∣ k+X^ik ∣ k−X^0jk ∣ k×X^ik ∣ k−X^0jk ∣ kt.

The mixing probability *μ*_*i*∣*j*_(*k*∣*k*) is given by

(14) $$\begin{array}{c} \mu_{ij}\left(\;k\;|\;k\;\right)=\frac{1}{\mu_{j}\left(k\;|\;k\right)}p_{ij}\mu_{i}\left(\;k\;|\;k\;\right), \end{array}$$

where the predicted mode probability *μ*_*j*_(*k* + 1∣*k*) = ∑_*i*=1_^*r*^*p*_*ij*_*μ*_*i*_(*k*∣*k*) and *p*_*ij*_ is the probability from *M*_*i*_(*k*) to *M*_*j*_(*k* + 1).

*(b) Kalman Filter.* In the Kalman filter framework, each KF/EKF would update the mixed state estimation with current measurement. The innovation covariance is given by

(15) $$\begin{array}{c} S_{j}=H_{j}{\tilde{P}}_{j}\left(\;k+1\;|\;k\;\right)H_{j}^{T}+R_{j}. \end{array}$$

We model the likelihood function for the matched filter as Gaussian pdf as the form:

(16) $$\begin{array}{c} \Lambda_{j}=\frac{1}{{\left(2\pi \right)}^{0.5}\sqrt{\left|S_{j}\right|}}\exp\left\{\;-0.5v_{j}^{T}S_{j}^{-1}v_{j}\;\right\}, \end{array}$$

where

(17) $$\begin{array}{c} v_{j}=z\left(\;k+1\;\right)-{\tilde{z}}_{j}\left(\;k+1\;|\;k\;\right). \end{array}$$

*(c) Mode Probability Update.* Once each model has been updated with measurement *z*(*k* + 1), the mode probability *μ*_*j*_(*k* + 1∣*k* + 1) is updated by mode likelihood Λ_*j*_ and the predicted mode probability *μ*_*j*_(*k* + 1∣*k*) with normalization factor *c* = ∑_*i*=1_^*r*^*μ*_*i*_(*k* + 1∣*k*)Λ_*j*_ is

(18) $$\begin{array}{c} \mu_{j}\left(\;k+1\;|\;k+1\;\right)=\frac{1}{c}\mu_{j}\left(\;k+1\;|\;k\;\right)\Lambda_{j}. \end{array}$$

*(d) State and Covariance Combiner.* The state estimation and associated covariance are combined using the updated mode probability:

(19) X^0jk+1 ∣ k+1=∑i=1rμijk+1 ∣ k+1X^ik+1 ∣ k+1P^0jk+1 ∣ k+1=∑i=1rμijk+1 ∣ k+1·P^ik+1 ∣ k+1+X^ik+1 ∣ k+1−X^0jk+1 ∣ k+1×X^ik+1 ∣ k+1−X^0jk+1 ∣ k+1t.

## 3. Evaluation

### 3.1. Experiment Setup

We use four distinct sets of biological data to evaluate the proposed method. The first two datasets are heat motion positon datasets using the Sonomicrometry system [30]. The last two are arrhythmia ECG signals from MIT-BIH arrhythmia database [31]. The 3D beating heart motion positions are collected on the heart surface locating at a point one-third of the way from the starting point of the* Left Anterior Descending (LAD)* artery. The difference between two Sonomicrometry datasets lies in the heartrate, in which one is normal heart beat rhythm with more “constant” period and the other one is varying heart rate dataset (we exert the external stimuli occasionally to obtain the arrhythmia-like heart motion). The more detailed heart motion measurement information could be referred to [27]. The reason we select ECG signal datasets is that the ECG signals not only share the similar dynamics with heart motion but also contain different types of arrhythmia, which provides different situations occurring in the beating heart surgery to evaluate the proposed method. The ECG dataset 107 m is Premature Ventricular Contraction (PVC) arrhythmia commonly occurring among people with or without heart disease. The dataset 202 m is with atrial fibrillation (AF) and atrial fibrillation aberrated beat arrhythmia, which are the most common irregular heart rhythm and occur most often in people with heart disease.

The ability of the NFAM to track the beating heart motion through the complexed situation and provide accurate state estimates is assessed using combinations of the two models described in Section 2. To bring out of the benefits of including more than one model in IMM estimator, the following comparative cases have been studied with the four sets of experimental data.

1. Only NAM using four datasets
2. Only UIM8 using four datasets
3. Only UIM12 using four datasets
4. IMM8 using four datasets
5. IMM12 using four datasets

We evaluate the tracking performance of the filter in each case regarding root mean square position error, $\chi_{nms}=\sqrt{\left(1/n\right)(\chi_{err1}^{2}+\chi_{err2}^{2}+\cdots +\chi_{errn}^{2})}$.

Under the KF/EKF framework, the parameters in the all three algorithms are tuned by minimizing the RMS errors using the first 10-second data in each dataset. Since NAM is frequency domain based method such that its number of frequency components is determined by observing the frequency response, namely, PSD then is modified by trial and error method. In UIM, the model order variable (8 and 12) is firstly determined by doubling the number of harmonies in PSD of the heart motion and then corrected by trial and error method. We select the forgetting factor in both algorithms close to 1 such that the memory horizon at least covers one respiration motion period. The process noise covariance in NAM is chosen within certain scalar factor such that the RMS prediction errors are minimizing. The initial mode probability *μ* is decided according to the rough guess of the possibility level corresponding to each mode in specific dataset. The switching probabilities *p*_*ij*_ are estimated based on the sojourn time.

### 3.2. Results

**Figure 2:**
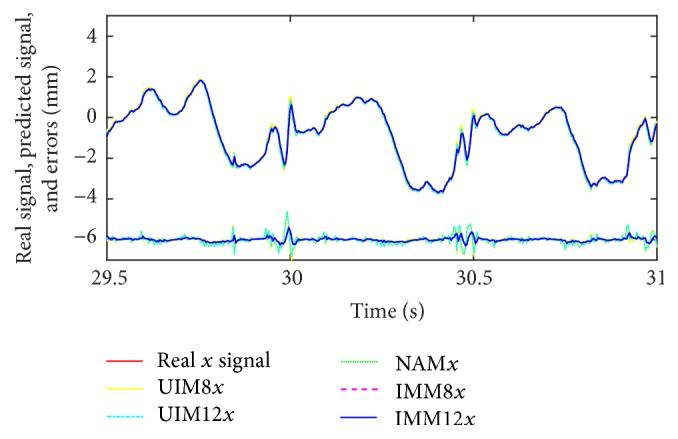
Long time scale prediction results of *x*-axis in heart motion constant dataset.

**Figure 3:**
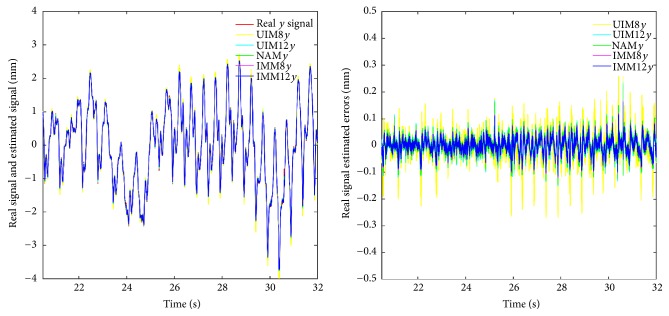
Middle time scale prediction of *y*-axis in heart motion varying dataset.

**Figure 4:**
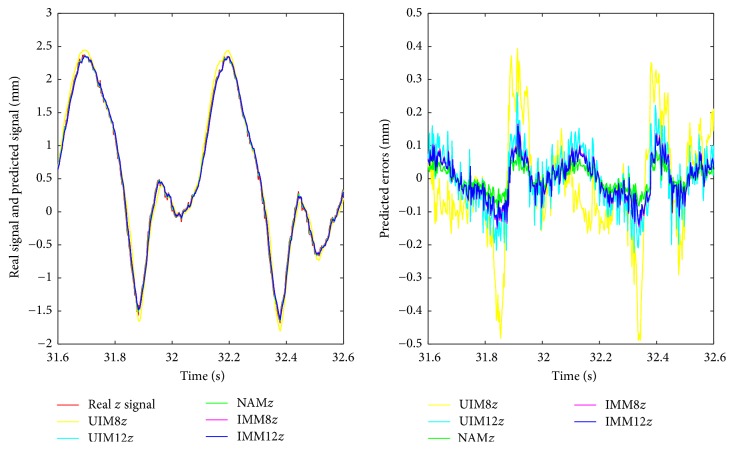
Short time scale prediction of *z*-axis in heart motion varying dataset.

**Figure 5:**
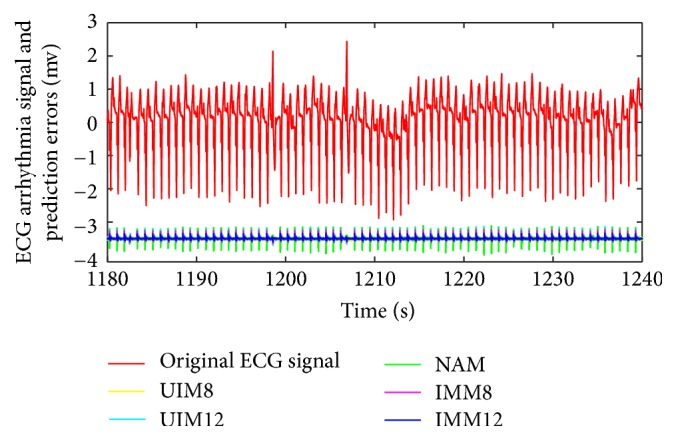
ECG PCV arrhythmia (107 m) signal and prediction error comparison.

**Figure 6:**
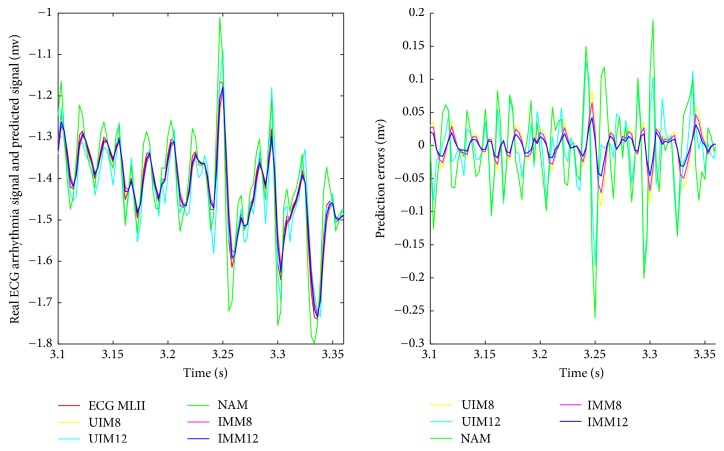
ECG AF arrhythmia (202 m) signal prediction short time scale results.

We summarize RMS prediction results and algorithm variance analysis in Table 1. We also present the comparative prediction curves in Figures 2–6.

### 3.3. Discussion

As the evaluation results shown, NFAM outperforms other two models regarding prediction RMS. In particular, NFAM has the capability to handle the situation where the diversity is severe and prior knowledge of the signal model is inadequate. We discuss the results through different comparative experimental scenarios.

*(1) NAM, UIM8, and IMM in Constant and Slowing Varying Heart Motion Datasets.* The “constant” beating heart motion preserves the inherited nonlinearity. We could observe that its trajectory pattern is relatively predictable over other chaos patterns. The quadratic term in the NAM is mainly used to account for the cardiorespiration interaction which is the most significant coupling existing in the normal cardiovascular signals, whereas UIM is lack of the quadratic term for this nonlinearity modeling. From Table 1 and Figures 2–4, we could clearly observe the prediction errors for constant dataset; IMM and NAM are almost at the same level and much better than UIM. For the slow varying heart motion case, IMM has lower prediction errors than NAM since the irregular pattern is beginning to show and pure nonlinearity is not the only dominant part anymore. 

*(2) NAM, UIM, and IMM in Two Arrhythmia ECG Datasets.* With the insufficient prior knowledge of the heart motion signal, we need to deal with the miscellaneous heart motion signals from the Coronary Artery Disease patients. IMM towers in all the algorithms with regard to the compensation of modeling uncertainties by using fused states from two models. Either NAM or UIM has limitation since its single model deficiency could not adaptively estimate all the states during all the regimes of the motion. NAM is worse at this situation due to the overfitting. In contrast, IMM has the mechanism to take into account the uncertainties. The prediction results in Table 1 and Figures 5 and 6 show the robustness of the IMM.

## 4. Conclusions

The proposed method is a self-adjusting variable-bandwidth filter, which is natural and suitable for target tracking task. The beating heart motion is complex and diverse such that it is necessary for the model to not only characterize the nonlinearity with small amount trajectory uncertainties but also cover the larger amount of uncertainties (arrhythmia-related pattern irregularities). We include the nonlinear handling model in IMM estimator along with the uncertainty adaption model to track beating heart motion. The motivation for combining two models is that agile target motion is likely to have more significant variations which single tracking model cannot adequately handle. After conducting the comparative experiments and discussion through the proposed ANM method, we conclude that we have improved beating heart motion prediction performance by dynamically fusing the states in the IMM framework covering both the nonlinearity description and model uncertainty dealing function. The more accurate and robust NFAM algorithm will allow us to expect improved tracking control performance with integration with model based control algorithm.

## Figures and Tables

**Table 1:** Prediction results of comparative methods.

Algorithms	Heart motion datasets		ECG arrhythmia datasets	
	Constant 3D RMS [mm] ± SD	Varying 3D RMS [mm] ± SD	107-V1 1D RMS [mv] ± SD	202-MLII 1D RMS [mv] ± SD
UIM8	0.587 ± 0.01	0.276 ± 0.01	0.036 ± 0.003	0.034 ± 0.003
UIM12	0.547 ± 0.01	0.152 ± 0.01	0.028 ± 0.004	0.029 ± 0.004
NAM	0.184 ± 0.01	0.102 ± 0.01	0.046 ± 0.003	0.053 ± 0.003
IMM8	0.195 ± 0.01	0.096 ± 0.01	0.024 ± 0.003	0.021 ± 0.003
IMM12	0.189 ± 0.01	0.093 ± 0.01	0.021 ± 0.003	0.018 ± 0.003
